# Supplementary material for: Are carbon emissions trading and green financial instruments synergistic? -Comprehensive quantitative research based on content analysis
Source: PLoS One. 2024 Mar 7;19(3):e0298601. doi: 10.1371/journal.pone.0298601 (PMC10919601; doi:10.1371/journal.pone.0298601)
Supplement: S1 Data — (ZIP) [file pone.0298601.s001.zip › Data/地级市Z_F工作报告.zip!/░▓╗╒/┬φ░░╔╜╩╨/└┤╫╘╣1⁄2╓┌║┼í╛┬φ┐╦╩2╛▌═°í┐_2010.docx]

# **政府工作报告（2010）**

【字体大小：[大](http://zwgk.mas.gov.cn/openness/detail/content/javascript:doZoom(18)) [中](http://zwgk.mas.gov.cn/openness/detail/content/javascript:doZoom(15)) [小](http://zwgk.mas.gov.cn/openness/detail/content/javascript:doZoom(14))】

——2010年1月6日在马鞍山市第十四届人民代表大会第四次会议上

市 长   周春雨

各位代表：

    现在，我代表市人民政府，向大会作工作报告，请予审议，并请市政协委员和其他列席人员提出意见。

****一、2009年工作回顾****

刚刚过去的2009年，是我市进入新世纪以来最为困难、极不寻常的一年，也是我们砥砺奋进、经受严峻考验的一年。面对国际金融危机给全市经济社会发展造成的严重冲击，在省委、省政府和市委的坚强领导下，市政府团结带领全市人民，认真贯彻落实党的十七大和十七届三中、四中全会精神，深入学习实践科学发展观，以庆祝新中国成立六十周年为强大动力，攻坚克难保增长，化危为机调结构，经济回升向好势头不断巩固，社会事业全面进步，民生改善成效明显，市十四届人大三次会议确定的主要目标任务全面完成，入围新中国成立六十周年60个“城市发展代表”行列，成为中部地区首个“全国文明城市”，在推进“科学发展上水平、率先全面达小康”征程上迈出了坚实步伐。

初步统计，全市实现地区生产总值650亿元，比上年增长12%；财政收入122.3亿元，增长11%；全社会固定资产投资546亿元，增长35%；社会消费品零售总额125.7亿元，增长18.6%；城市居民人均可支配收入突破2万元，增长11.3%；农民人均纯收入突破8000元，增长10.5%。全面小康实现程度达94.2%，比上年提升2.9个百分点。总体上看，在支柱产业遭受前所未有冲击的情况下，全市主要经济指标总量和人均值继续位居全省前列，增幅高于长三角城市平均水平，经济发展取得的成绩超出预期。

（一）突出保增长首要任务，经济回升向好势头不断巩固。面对严峻复杂的经济形势，市政府全面分析，准确判断，果断决策，从容应对，快节奏、高效率、大力度推进保增长各项工作。

扎实开展“企业帮扶年”活动，全面落实六大类85项帮扶措施，建立领导干部企业帮扶联系点制度，成立5个帮扶工作组，对大企业实行一企一策和一对一联系帮扶，对中小企业实行点面结合和分类扶持，想方设法帮助各类企业摆脱困境、渡过难关。积极搭建银企对接平台，完善小企业贷款风险补偿和奖励办法，努力破解企业融资难题。创造性地落实宏观调控减免税费政策，累计为各类企业减免税21亿元，减免费2.6亿元，缓缴税费2.2亿元；同时争取中央和省支持11.3亿元，本级财政安排3.1亿元，合计对企业各类补贴达14.4亿元。加强创业载体建设，完善创业服务体系，全年新发展个体工商户9631户；新发展私营企业1913户，总数突破1万户，个私经济纳税额达20.8亿元。全市企业在决战危机中强化自身，广大职工与企业同舟共济，为经济整体回升向好做出了突出贡献。马钢紧紧围绕“聚焦品种质量、强化内部管理”工作主题，建立市场快速反应机制，灵活调整生产经营策略，深入推进降本增效，在极其困难条件下保持了生产经营基本稳定。星马集团突出产品高端定位，产销重型卡车1.8万辆、专用汽车7000辆，销售收入突破70亿元。立白日化、雨润食品等外来投资企业生产经营快速攀升，泰尔重工等一批中小企业产销两旺。全年新增规模以上工业企业103户，规模以上工业实现增加值352亿元，增长14.5%。

把重点项目建设作为保增长、增后劲的关键举措，健全市级领导牵头调度制度，强化项目协调推进机制，实行重点项目动态调整，全方位增加有效投入。重点项目完成投资213亿元，省“861”计划项目完成投资居全省前列。科达机电、达利食品、稳润光电等52个项目建成投产，天成纺织搬迁扩建等一批项目加快推进，华菱3万辆重卡等90个项目开工建设，一电厂2台60万千瓦机组“上大压小”扩建项目即将获批。结合马钢周边环境整治和金家庄老工业区搬迁，强力推进马钢“十一五”后期结构调整规划项目前期工作，累计征迁住户2397户、企业44家，征迁面积33万平方米。抢抓国家扩大内需政策机遇，争取中央新增投资项目81个、资金3.4亿元。着力加强与央企对接合作，总投资672亿元的23个项目达成合作意向。精心谋划战略性产业项目，总投资1180亿元的32个项目充实重大项目库。

（二）加快经济发展方式转变，结构调整迈出实质性步伐。充分利用国际金融危机形成的倒逼机制和洗牌效应，坚持自主创新与结构调整相结合，加速推进产业结构优化升级。大力扶持发展新兴产业，引进上海晟峰、东华软件、美国微因等国内外知名软件、动漫企业80多家，一批新兴产业呈现蓬勃发展势头。成功跻身国家知识产权试点市行列，新认定国家火炬重点高新技术企业2家、国家高新技术企业21家，总数分别达6家、83家；新增省级高新技术产品26个，总数达155个；全市实现高新技术产业产值360亿元。马钢成为第三批国家级创新型试点企业，十七冶被确定为球团机械设备工程安装验收规范国家标准主编单位，矿院研制的新型磁选机被认定为国家首批自主创新产品，中冶华天“合金化微调方法”填补了我市海外专利申请空白。质量兴市战略深入实施，国家钢铁及制品质量监督检验中心加快建设，省机床及刃模具产品质检中心成功落户。国家节能重点项目建设加快推进，马钢尘泥脱锌转底炉等项目建成投产，我市成为全国再生资源体系建设试点城市。全市万元地区生产总值综合能耗下降5.5%，主要污染物排放量控制在省下达目标以内。

服务业提升发展。依法彻底解决多年困扰大华国际广场二期项目建设的拆迁难题，八佰伴大型商贸购物中心和香港城百货市场入驻运营。法国欧尚、南京金鹰、上海百联等一批知名商贸企业签约落户，核心商圈集聚效应明显增强。合理引导住房开发建设与消费，房地产开发投资平稳增长，新建和二手商品房交易量分别增长1.3倍和1倍，住房公积金支取使用和贷款刷新纪录，房地产市场健康发展。加大旅游资源开发力度，龙湾水文化度假主题公园成功落户，皇冠假日酒店建成开业，一批生态旅游园投入运营，全年旅游业总收入增长17.5%。

（三）深入推进改革开放，经济社会发展活力进一步增强。全面实施新的土地征迁补偿安置办法，严格实行国有建设用地净地出让制度，着力破解项目“落地难”问题。市政府机构改革稳步实施，事业单位岗位设置管理纵深推进。深化市直行政事业单位国有资产管理改革，为实施产权集中管理、最大程度发挥国有资产效益创造了条件。按照“整合资源、运行高效、统一监管、节约成本”的原则，全市集中统一的招标采购监管体制初步建立。教育管理体制逐步完善，15所市属义务教育阶段学校和2所幼儿园移交区级管理，义务教育学校绩效工资改革平稳实施。深化医药卫生体制改革全面展开，基层医药卫生体制综合改革试点快速推进，跻身全国公立医院改革试点市行列。成为全省人口与计划生育综合改革试点市。成品油价格和税费改革平稳实施。工商系统12个集贸市场办管脱钩全面完成。

金融创新亮点纷呈。截至上年底，各金融机构存款余额696亿元，增长26.4%，当年新增145亿元。本地金融机构贷款余额461亿元，增长40.5%；累计发放贷款770亿元，当年新增133亿元，超过前3年总和。同时，全市金融投放呈现多元化、多渠道、多品种新格局，票据融资192亿元，异地银行在我市发放贷款77.5亿元，资本市场累计融资230多亿元，企业市场融资总额突破1200亿元，实现历史性跨越。全国首家以地级市组建的马鞍山农村商业银行挂牌，发起设立的当涂新华村镇银行揭牌开业。浦发银行马鞍山支行开业运营。总规模30亿元的皖江城市带承接产业转移投资基金正式设立，成为全省首个产业基金。以政府投资公司为融资平台，在全省第一个面向全国成功发行10亿元理财产品，有力支持了县区和开发区加快发展。1.5亿元中小企业集合债券成功发行并投入使用，1.1亿元中央发行的地方政府债券有力支持了经济和社会发展薄弱环节，20亿元城市建设债券发行前期工作稳步推进。全省注册资本规模最大的小额贷款公司顺利组建，3家小额贷款公司开业，2家担保公司注册资本金增至亿元。首期注册资本5000万元的安徽省高新创业投资公司正式挂牌运营。企业上市融资和再融资工作实现新突破，泰尔重工、鼎泰稀土一次性通过证监会发行审核，星马汽车重组华菱重卡整体上市加快推进，山鹰纸业可转换债券转股顺利实施，奥盛钢缆、华菱西厨、惊天液压上市前期工作扎实开展。

对外开放不断扩大。建立市领导招商联系点制度，组建6个招商分局和18个招商小分队，加快构建信息、利益共享机制，招商引资工作体制机制进一步完善。精心组团参加第四届中博会暨第五届国际徽商大会，成功举办马鞍山（杭州）土地推介招商会、中国（马鞍山）刃模具暨机床博览会，赴香港、广东、福建、上海、浙江、江苏等地招商活动取得重要成果。全年实际利用外资6亿美元，增长17.4%；实际利用内资260亿元，增长15%。市经济开发区南区路网框架进一步拉开，30个项目建成投产，新增销售收入超亿元企业3户，全区实现财政收入6.7亿元。慈湖经济开发区省级台湾工业园挂牌，台湾和桐化工等一批项目落地建设。市经济开发区和慈湖经济开发区双双跻身全省省级开发区十强行列。建立中小企业出口风险补偿资金，实行政府主管部门推荐、平台公司统一担保、银行审核放贷的融资保障机制，有力促进了中小企业出口逆势上扬。全年完成进出口总值16亿美元；实现外经营业额1.85亿美元，增长75%。口岸正式对外籍船舶开放，边防检查站组建挂牌，保税仓库和出口监管仓库按期建成，外贸进出口服务保障体系基本形成，对进一步增强城市服务功能、扩大对外开放、提升区域发展地位产生了重大而深远影响。口岸直运量实现翻番，外运量占全省份额80%以上。积极参与皖江城市带承接产业转移示范区规划编制，一批重大产业和基础设施项目纳入国家规划，南部沿江承接产业转移集中区前期工作稳步推进。与宿州市开展结对合作、与巢湖市跨江联动发展取得积极成果。

（四）县区综合实力不断攀升，城乡一体化进程扎实推进。当涂县实现地区生产总值145亿元，增长15%；完成财政收入18亿元，增长15.5%；长江钢铁精品钢技改等一批项目开工建设；博望镇被授予“全国文明乡镇”称号，扩权强镇试点工作扎实开展。实施新的市区财政管理体制，将1766户市级企业财税关系下放区级管理，三区财政收入均突破6亿元，增幅均超过30%，自主发展能力明显增强。花山区软件产业发展势头良好，软件大厦和晟峰集团安徽总部大楼加快建设，花山美居项目基本完工，省级花山经济开发区筹建工作取得重要进展。雨山区视聆通游戏动漫产业基地初具规模，国家级再生资源集散市场开工建设，安民农副产品交易中心二期即将投入运营。金家庄区金属制品产业集群规模持续扩大，和菱包装新基地等重点项目建成投产，区域物流服务功能和效益进一步显现。与此同时，三区共计征迁土地面积超过2万亩，拆迁房屋面积近100万平方米，为全市发展大局作出了重要贡献。

城乡一体化发展深入推进。花山区张庄中心村一期基本完工，霍里镇“镇改街”全面完成。新农村“四条示范线”启动建设，试点镇村示范作用明显。全年完成小城镇建设投资14.1亿元。建成农村公路80公里。一五圩国家大型排涝泵站改造工程顺利竣工，5座病险水库、34座涵闸除险改造全面完成。优化调整农村学校布局，留守儿童和外来务工人员子女教育得到加强。在全省率先实现文化信息传输“村村通”，新建农家书屋39个。14个乡镇综合文化站建成使用，用1年时间完成了国家和省确定的5年达标任务。农村有线电视“进村入户”工程加快实施，乡镇综合信息服务站实现全覆盖。提前2年完成乡镇卫生院标准化建设任务，53所标准化村卫生室建成使用。

现代农业稳步发展。出台落实政策措施，加快发展现代农业，促进农业增效、农民增收。全年实现农业增加值25.1亿元，增长5.3%。总投资4.7亿元的当涂现代农业示范园启动建设。当涂县被列入“中国1000亿斤粮食增产计划项目县”，成为“中国生态养蟹第一县”。推进和规范农村土地承包经营权流转，流转面积达17.6万亩，占全市承包耕地面积的24.8%。50亩以上粮食规模种植户达561户，全市粮食总产再创历史新高。畜禽规模养殖比重达50%，水产生态养殖比重提高到76%。农业产业化超亿元龙头企业增至10户，其中超10亿元企业2户。新增农民专业合作社80家，总数达177家。“三资”农业加快发展，全年到位资金4.9亿元。主要农作物政策性农业保险参保率达94.5%，居全省首位。粮食市级储备进一步充实，新农村科学储粮示范工程走在全省前列。

（五）城乡建设大规模展开，城市功能和环境质量明显提升。抓住国家扩大内需和建设成本相对较低的有利时机，全面开工建设了一批投入较大、多年想干而没有条件干的重大基础设施项目，城乡建设投资首次突破百亿元，达105亿元。长江公路大桥主体工程加快建设，斜拉桥、引桥及接线工程全面开工，分三年实施的长江江心洲段河道整治工程全面启动。宁安城际铁路站房建设方案得到优化，沿线征迁同步快速推进。完成东环路股权回购，顺利实现资产交接，高速化改造工程正式动工；对部分路段实施高架改线，为主城区加速东扩开辟了无障碍通道。马濮旅游大道竣工通车，与江苏对接的马濮一级公路改扩建工程开工建设，宁马边界国道收费站成功合并。天顺码头、长江港口码头投入运营，港口服务功能进一步提升。

城市基础设施建设大力度推进。高标准完成湖南路、佳山路、花雨路“白加黑”改造及综合整治，慈湖河路北段、湖南东路、江东大道三期建成通车。湖东南路加快推进，沿江大道北段、九华东路开工建设。按照“尊重历史、面向未来，有利交通、民主决策”的原则，对多年来市民广为关注的团结广场及周边进行改造，整体效应初步显现。雨山湖公园南门入口改扩建工程全面完工，滨湖公园开工建设。12个电网对接项目加快实施，园水变电站等项目建成运营，高压线廊整合移位改造启动实施，供电安全性、可靠性和线廊集约用地水平明显提高。与安徽电信、移动、联通分别达成战略合作框架协议，数字城市建设有序推进。

城乡环境建设迈上新台阶。慈湖河流域生态环境综合整治取得阶段性成效，上游河道清淤全面完成，水土保持与生态恢复同步推进，中下游河道治理工程启动建设。市经济开发区污水处理厂二期、当涂污水处理厂一期竣工运行，慈湖污水处理厂、东部污水处理厂基本完工。深入开展水环境治理，永丰河等水系整治顺利完成。向山垃圾处置场二期、尾矿库酸水处理工程相继完工，渗沥液处理改扩建工程开工建设。城市人居环境持续改善，通过国家园林城市复查验收，生活质量竞争力名列全国第9位。

城乡规划管理水平稳步提高。土地利用总体规划修编被列入国务院审批范围，规划大纲通过国土资源部批准，城市总体规划修改工作基本完成。实施巩固全国文明城市成果三年行动方案，城市公共文明指数测评取得好成绩，名列全国参评地级市第4位。启动农村集体土地确权工作，集体土地上房屋权属登记发证加快推进。突出标本兼治，加大疏导力度，一批摊群点专业市场建成使用。户外广告、门头招牌设置进一步规范，建筑垃圾、渣土运输管理得到加强。严格实行土地出让“招拍挂”制度，全面加大土地出让金清欠追缴力度，扎实开展清理整改土地违法违规行为专项行动，依法依规妥善处理土地历史遗留问题。

（六）着力保障和改善民生，社会建设和管理全面强化。投入资金近10亿元，深入实施50项民生工程，惠及全市城乡居民。优先发展城市公共交通，补贴近1500万元更新70辆高档公交车并将分批投入运营，提升改造公交场站，调整、延伸和增加公交线路，在所有线路落实特殊群体免费乘车政策，并规范管理公交广告，努力使公交成为城市文明的流动风景线。完成新建3万套安置房规划选址，661套廉租住房竣工交付使用。金家庄部分地区居民搬迁步伐加快，江边地区1800户居民基本搬迁完毕。农村敬老院新增床位1200余张，五保集中供养率超过40%，贫困残疾群体帮扶救助力度进一步加大。

就业和社会保障工作扎实推进。出台落实“五缓、四降、三补贴、两协商”等稳定就业10项政策，集中开展“就业服务月”活动，加强职业技能培训和创业培训，启动失业预警机制，积极防范和化解就业矛盾。大力推动以创业促就业，成为全国首批创业型试点城市。全年新增城镇就业3.4万人，援助困难人员再就业4500人，农村劳动力转移就业2万人，完成各类人员培训2.3万人次，城镇登记失业率控制在3.3%以内。在全省率先整合市辖区新农合与城镇居民医保政策，实现定点医院、报销比例、医药目录并轨，与上海建立常居异地退休人员医疗保险结算服务制度。实施居民生育保险制度，成为全国居民生育保险工作试点市。城乡低保标准提升至全省首位，实现市区农村和城镇、当涂县城镇和市区“两个统一”，政策差别逐步消除。进一步提高新农保基础养老金和被征地农民养老保险待遇，建立丧葬抚恤金制度，广大农民参保积极性明显提高。

公共服务水平逐步提升。中小学校舍安全工程稳步实施。二十中迁址主体工程封顶，南部高中前期工作加快推进，石桥中学进入省示范高中行列，中等职教园区启动建设，高中阶段教育毛入学率达86.8%。安工大东校区、河海大学文天学院、马鞍山职业技术学院续建工程扎实推进。实施义务教育民办学校补助制度，对高校和中职学校困难家庭学生实行资助。市博物馆、图书馆建成并免费向市民开放，硬件设施居全国中等城市前列。大剧院主体工程完成，集科技馆、青少年宫和妇女儿童活动中心为一体的综合馆即将开工建设。太白楼维修暨李白纪念馆陈列改造完工，谪仙园建成开放。成功举办’2009马鞍山中国李白诗歌节暨国际吟诗节，《唐诗三百首》特种邮票在我市首发，文化艺术作品创作、文化遗产保护、城市历史文化和李白研究成果丰硕，文化工作受到国家文化部表彰。科技进社区进乡村工程成效明显。市档案馆改扩建工程按期完成并投入使用。《马鞍山市志》（1988-2005）编纂完成，《太平府志》点校出版。人民医院门诊大楼主体工程封顶，中医院南院建设加快推进。人口计生工作抓基层强基础、抓队伍强素质取得积极成效，建成“一站式”婚育服务中心，人口出生率控制在10.8‰以内。市体育中心前期工作基本完成，学校体育场所免费向社会开放，社区体育设施基本实现全覆盖，我市运动员在十一届全运会上取得3金4银好成绩。第二次全国经济普查工作受到国务院检查组表彰。调整城乡义务兵家庭优待金发放标准，大幅提高退役士兵自谋职业一次性经济补偿金，民兵训练基地建成使用，兵役和国防动员工作扎实有力。民族工作受到国务院表彰，宗教事务管理进一步加强。人防事业不断推进。国家一级气象站建成。对台经贸和文化交流增多。老年教育条件得到改善、工作成效明显，青少年工作和妇女儿童事业取得新进展。

顺利完成国庆60周年安保各项任务，社会保持和谐稳定。广泛深入开展领导接待日、包案带案下访和“信访积案化解年”活动，一批重点信访案件得到有效解决，去省进京上访总量明显下降。加强社会治安综合治理，严密治安防控措施，校园周边环境专项整治和“重点工程创平安”活动扎实开展。社区矫正新体制基本建立，安置帮教、法律援助和人民调解工作取得新成效。完成社区规模调整和第四届社区居委会换届选举，和谐社区、特色社区创建取得积极成果，半山花园社区荣获“全国文明社区”称号。切实保障人民群众身体健康和生命安全，甲型H1N1流感、手足口病防控取得明显成效，食品药品安全放心工程深入实施。“安全生产年”活动扎实开展，全市安全生产形势保持平稳。应急管理工作得到加强，预防和处置各类突发事件能力进一步提升。

（七）深入践行科学发展观，政府自身建设得到加强。根据市委统一部署，政府系统围绕“科学发展上水平、率先全面达小康”主题，突出保增长、保民生、保稳定最大实践，扎实开展深入学习实践科学发展观活动。全面分析检查制约发展的突出问题，落实87项具体整改措施，组织开展“回头看”，进一步增强了贯彻落实科学发展观的自觉性和坚定性。

一年来，市政府坚持党的领导，坚决贯彻落实市委各项决策部署。认真执行市人大及其常委会决议、决定，定期向市人大常委会报告工作、向市政协通报情况，主动接受市人大的法律监督、工作监督和市政协的民主监督。政府系统承办的12件人大议案、127件建议批评意见、260件政协提案全部办复。制定法治政府定性定量指标体系，全面规范行政处罚自由裁量权，行政复议委员会试点工作扎实开展。建立完善依法行政制度，科学、民主、依法决策机制进一步健全。

巩固行政审批“两集中、三到位”工作成果，实施行政审批流程再造，推行并联审批，审批效率明显提高。健全政务公开制度体系，推动行政权力公开透明运行。电子政务统一平台投入使用。政务督查力度加大，目标管理体系进一步完善。深入开展政风行风和效能评议，11名违反工作岗位禁令的直接责任人受到严肃处理，损害群众利益的突出问题得到及时纠正。加强对新增中央投资项目监督检查，实行政府投资重点项目和民生工程派驻监察，严肃查处工程建设等领域违法违纪行为。“小金库”专项治理深入开展，矿产资源生产秩序和经营活动监管进一步加强。认真落实中央厉行节约八项要求，公务接待费用支出在上年基础上削减10.6%，车辆购置及运行费用、公款出国（境）经费支出在近3年平均数基础上分别降低23.8%、20.3%。充分发挥审计监督作用，预算执行、政府投资建设项目和经济责任审计取得积极成效。

各位代表，由于特殊的产业结构、企业结构和较高的外贸依存度，过去一年我市受到国际金融危机的冲击是强烈的，经历的考验是严峻的，面临的困难是空前的，承受的压力是巨大的。即便如此，全市经济平稳较快增长势头没有下行而是进一步上扬，城乡建设力度没有减小而是进一步加大，城乡居民收入水平没有降低而是进一步提高，全面小康进程没有放缓而是进一步加快，广大干部群众精神状态没有消沉而是进一步激昂。成绩极为不易，经验弥足珍贵。这是省委、省政府和市委正确领导的结果，是全市上下同心协力、顽强拼搏的结果。在此，我代表市人民政府，向辛勤工作在各条战线上的广大干部群众，向给予政府工作大力支持的市人大代表、市政协委员，各民主党派、工商联和无党派人士，各人民团体，致以崇高的敬意！向参与、支持马鞍山现代化建设的中央和省驻马单位、解放军和武警官兵，境内外投资者、海内外朋友，表示衷心的感谢！

在充分肯定成绩的同时，我们也清醒认识到，我市经济社会发展还面临不少困难和挑战，政府工作中还存在一些不足和问题。受国际金融危机的影响，我市经济结构不优、产业层次不高、自主创新能力不强的深层次问题“水落石出”，推动经济结构调整、加快转变发展方式任重道远；项目引进难、拆迁安置慢，大项目和好项目不多，成为制约经济社会发展的瓶颈；中心镇规模偏小、实力较弱、基础设施不完善，尚未真正形成特色鲜明、错位发展的城镇格局；广大群众关注的教育、医疗、就业、环境等领域的问题不同程度存在，政府社会管理和公共服务水平有待提高；少数工作人员责任心不强、工作作风不实、服务水平不高，消极腐败现象还时有发生。这些问题，已经引起我们高度重视，正在采取有力措施加以解决。

****二、2010年主要目标及重点工作****

今年是实施“十一五”规划的最后一年，也是巩固经济回升基础、谋划“十二五”发展、加速全面小康进程的关键之年。做好今年经济社会发展工作，具有十分重要的意义。

从总体上看，今年我市的发展环境将好于去年。中央宏观调控政策“总体稳定、稳中有调”，继续实施积极的财政政策和适度宽松的货币政策，为我们在发展中促转变、在转变中促发展创造了诸多有利条件。国家全面启动皖江城市带承接产业转移示范区建设，沿海发达地区经济加速转型和调整生产力布局，有利于我们发挥综合比较优势，抢占后危机时代的发展先机。加之我市去年谋划和新开工了一大批项目，以及通过对接央企和招商引资成功签约了一批项目，为今年投资较快增长奠定了基础。特别是去年底召开的市委七届十二次全会，立足新起点，树立新标杆，明确新思路，为当前和今后一个时期发展指明了方向，绘就了蓝图，预示着全市经济社会新一轮更高更大更快发展的时期已经到来。但我们也要看到，当前国际金融危机余波未尽，经济运行尚未根本好转，发展的复杂性不容低估。尤其令我们警醒的是，省内兄弟城市呈现出百舸争流的竞相发展态势，已将我市置于不进则退、慢进也是退的紧要关口。如何巩固在全省东向发展中的主体地位、主体优势，更好地发挥主体作用、主体贡献，做到盯住标兵、拉开追兵，将是我们面临的最大挑战、最大考验。我们务必要全面辩证地把握形势，始终保持清醒的头脑，始终坚定必胜的信心，积极拼抢机遇，勇于突破难点，提高度，扩气度，加速度，不断开创经济社会发展新局面。

做好今年各项工作，我们要全面贯彻落实党的十七大和十七届三中、四中全会、中央和全省经济工作会议、市委七届十二次全会精神，以邓小平理论和“三个代表”重要思想为指导，深入贯彻落实科学发展观，按照“提高质量、做大体量、扩展数量”的要求，紧紧围绕“坚持科学发展、走在全省前列”的总体思路，把巩固和增强经济回升向好势头与加快经济发展方式转变有机统一起来，把深入开展“项目推进年”活动作为促发展、调结构的重要抓手，把推进改革开放、自主创新和城乡一体化作为经济社会发展的强大动力，把保障改善民生、促进社会和谐作为加快发展的最终目的，努力实现经济社会更高水平、更大体量、更快速度发展，全面完成“十一五”规划目标。

综合各种因素，今年经济社会发展的主要预期目标为：地区生产总值增长13%以上；财政收入与地区生产总值同步增长；全社会固定资产投资增长30%；社会消费品零售总额增长15%；城市居民人均可支配收入增长10%；农民人均纯收入增长8%；城镇登记失业率控制在4%以内；万元地区生产总值综合能耗下降4%；主要污染物排放量、人口自然增长率控制在省下达指标以内。实现上述目标，我们将着力抓好以下工作：

（一）聚焦结构调整主线，加快转变经济发展方式，构建“6653”现代产业体系。调整经济结构、转变发展方式，是今年经济工作首要任务，对马鞍山这样一个经济结构、产业结构特殊的城市来说，显得尤为重要而迫切。要结合现有产业基础和条件，着力构建“6653”现代产业体系，加快形成现代加工制造基地、高新技术产业基地、区域性特色化现代服务基地。

****做大做强六大优势主导产业。****依托现有骨干龙头企业，加快技术改造步伐，着力提升钢铁制造、汽车制造、装备制造、电力能源、精细化工、食品加工6大优势主导产业的核心竞争力，努力形成一个超千亿级、五个超百亿级的产业。支持马钢加快结构调整步伐，尽快实施配套项目建设，促进当涂县钢铁产能置换项目加紧实施。做强重卡和专用车制造业，大力引进发展整车及核心零部件项目。整合装备制造业资源，提高大型成套设备生产能力。推动现有电力重点项目建设，积极打造皖电东送前沿基地。充分挖掘现有企业生产能力，着力延伸上下游产业链，促进精细化工产业快速壮大。实施食品加工扩能改造，引进更多的食品加工龙头企业入驻我市，建设长三角食品加工基地。

****培育壮大六大新兴产业。****坚持有所为有所不为，强化政策集中支持，引进和培育一批领军龙头企业，加快发展新材料、新光源、新能源、节能环保、生物医药、电子信息6大新兴产业。坚定不移地把自主创新作为经济社会发展的主战略，进一步完善和落实自主创新政策，开展创新企业培育和试点示范活动，着力壮大一批拥有自主知识产权的高新技术企业。支持矿院金属矿山开采安全与健康国家重点实验室和技术服务平台项目，深入实施与清华大学、中国科技大学、中科院合肥物质研究院产学研合作，充分发挥安工大等市域内高校培养各类新型人才的作用，集成科技资源推进创新创业创意载体建设，努力让更多的科技资源在我市集聚，促进更多的科技成果转化及产业化。确保全年新增高新技术企业15家、高新技术产品30个以上。

****改造提升五大传统优势产业。****坚持外延扩张和内涵提升并重，大力发展规模经济，加大企业技术改造力度，推动产品向系列化、品牌化、高端化发展，进一步改造提升造纸印刷、金属制品、机械加工、纺织服装、农产品加工5大传统优势产业。与此同时，落实促进建筑业加快发展的政策措施，不断提升建筑业发展规模和水平。实施万能达燃煤机组系统优化等7个重大节能工程，促进低碳经济、循环经济、绿色经济加快发展。

****加速发展三大现代服务业。****围绕完善城市综合服务功能，重点发展生产性服务业、生活性服务业和面向农村的服务业。加快长运物流港、华东物流园和长江大桥仓储物流园建设，推动软件动漫及服务外包成为新的经济增长点，促进生产性服务业与现代制造业有机融合。建成沃尔玛购物广场，开工建设欧尚大型购物超市，实施金鹰、杉杉现代商业项目，提升现代商贸发展水平，引导消费结构升级。大力培育跨区域、广辐射、上档次的品牌专业批发市场，加快特色街区建设，完善社区商业便民服务体系。继续做好家电、汽车、摩托车下乡工作，进一步完善农村商品流通网络，改善农村消费环境，挖掘农村消费潜力。综合开发滨江和濮塘旅游资源，加快建设龙湾水文化度假主题公园等现代大型旅游项目，不断壮大旅游业规模，提升旅游业层次。建立健全服务业统计和考核制度，为经济转型升级和服务业加快发展提供决策依据。

（二）大手笔展开城乡规划建设，进一步拉开城乡融合发展大框架，倾力打造城市新形象。

****强力实施“1255”城市发展战略。****树立全域马鞍山理念，着力拉开城市框架，拓展城市发展空间。按照“提升品位、完善功能”的要求，做大做优主城区。规划建设滨江新区和秀山新区，力争到“十二五”末，整体建成，展示新貌。本着“科学规划、基础先行”的原则，着手打造姑孰和博望2个副城区，加快构建连接市区和副城区的高水平快速通道。遵循“产业特色、扩权强镇”的思路，加快建设向山、石桥、黄池、太白、年陡5个中心镇，使之成为全市加快发展的生力军。强化“合理布局、错位发展”的理念，着力打造马鞍山经济开发区、南部沿江承接产业转移集中区、慈湖港口物流加工区、江心洲生态旅游区、濮塘休闲度假区5个产业功能区，使其成为承接产业转移的重要载体，努力构建跨越发展的重要增长极。

****全力加强城市基础设施建设。****扎实推进马鞍山长江公路大桥建设，完成长江江心洲段河道整治一期工程。加快宁安城际铁路土地征迁步伐，积极配合、协调推进铁路主线及站场建设。强力实施东环路高速化改造，基本完成原址路段改造主体工程，启动改线路段高架工程建设。完成马濮一级公路改扩建工程，打通至禄口国际机场快速通道。加快县乡公路改造步伐，认真做好205国道、313和314省道市政化改造以及黄池大桥项目前期工作，早日实现中心镇15分钟上高速的目标。合理利用港口岸线资源，全面提升港口吞吐能力，大力发展水运事业。集中力量打造城市东部路网，新建星光大道、东部环路、丰收路和桥山路，续建霍里山大道、九华东路。完善城市南部路网，实施采石河路高水平市政化改造，建成湖东南路。推进沿江大道建设，贯通红旗北路，续建钟山路、泰山路和勤俭路拓宽工程，实施湖西路、艳阳路“白加黑”综合改造。开工建设江东大道地下人行通道，缓解市二中周边交通压力。深入落实优先发展城市公共交通战略，加快实施高压线廊整合移位改造，推进川气东送工程管线建设，进一步完善供水、通讯等公共设施。

****着力优化城乡环境。****巩固提升全国文明城市创建成果，扎实做好迎接国家环保模范城市复查验收、国家卫生城市暗访检查工作。实施东环路与采石河路、葛羊路2个城市出入口环境综合整治，努力提升城市形象。完成马濮旅游大道绿化配套建设，使之成为又一条高水平景观大道。建成滨湖公园，实施南湖公园及花雨广场景观提升工程，启动东湖、秀山湖公园建设。赋予城市建设更多文化内涵，努力实现建筑风格与人文环境有机融合。继续推进慈湖河流域生态环境综合整治，重点实施中下游河道治理和沿河绿化景观工程，实现三年整治目标。续建东部污水处理厂和慈湖污水处理厂，新建开发区南区污水处理厂。开工建设危险废弃物集中处置中心，启动实施污水处理厂污泥处置工程。加强林业生态重点工程建设，统筹做好城乡绿化工作。实施恒兴、西塘等水系整治，加强农村水环境污染治理。进一步完善环卫设施，建成向山垃圾场渗沥液处理改扩建工程，开工建设新的建筑垃圾处置场和有机物处理中心。规范小区物业管理，推进城市管理数字化，进一步健全和落实城市管理长效机制。

（三）坚定不移地实施大开放主战略，主攻招商引资头号工程，推动开放型经济再上新台阶。

****高强度推进招商引资工作。****招商引资是决定马鞍山未来发展的关键所在。去年底以来，全市上下认真落实市委、市政府决策部署，开门大招商、落地倍亲商，招商引资力度空前加大、氛围日益浓厚。我们要巩固当前好势头，持续升温，乘势而上，锲而不舍，让招商热潮一浪高过一浪，真正使招商引资成为扩大有效投入的重要支撑，成为转变发展方式的重要途径。突出重点区域，坚持以我为主、择优选择，有针对性地开展专题招商、产业招商，着力引进投资额度大、科技含量高、产业关联度强的基地型、龙头型大项目、好项目，进一步提高招商实效。坚持领导带头招商、招商分局驻点招商和小分队专业招商相结合，鼓励以商招商、全员招商，努力形成人人关注招商、个个投身招商、全面推进招商的浓厚氛围。加大招商引资协调调度力度，健全项目商谈、签约、落地机制，出台落实考核奖惩办法，促进招商引资持续健康发展。充分利用上海世博会平台，进一步扩大城市影响，展示城市形象，增强对外来投资的吸引力。切实做好外事、侨务和对台工作，在贯彻落实有关政策要求的同时，发挥其在招商引资中的积极作用。力争全年实际利用外资7亿美元、内资320亿元以上。

****全力推动园区建设和发展。****加快推进市经济开发区扩区升级规划及实施工作，积极创建省级高新技术产业开发区，争取在招大引强上实现明显突破。推动慈湖经济开发区“一区多园”发展，强化要素保障，增强优势产业集群效应。整合南部沿江发展空间，加紧展开承接产业转移集中区建设相关工作，精心打造若干特色园区，力争在示范区建设中发挥先导作用。拓展当涂经济开发区发展空间，加快花山经济开发区建设，支持雨山区、博望镇申报筹建省级开发区，促进金家庄工业园特色产业发展壮大。明确功能定位，规划建设江心洲生态旅游区。高水平推进濮塘风景区及周边地区组团发展，积极培育和发展濮塘休闲度假区。坚持集约节约用地，鼓励企业建设多层厂房，着力提高单位土地面积投资强度，增强园区可持续发展能力。

****大力促进对外经贸合作。****立足国际国内两个市场，在更大范围、更广舞台、更高层次上参与经济合作交流。加快集装箱外贸内支线定期航班开通步伐，增加航班密度，促进更多企业在我市口岸报关直运。抓住国际市场回暖机遇，加快出口基地培育和建设，进一步转变外贸增长方式，促进出口稳步提升。切实发挥中小企业出口融资政策引导和保障功能，及时为有市场、有订单、有效益的企业提供融资便利，确保企业不因资金困难影响出口。全年外贸进出口总额增长20%以上。鼓励十七冶、中冶华天等优势企业加快“走出去”步伐，开展境外工程总承包，参与援外工程建设，带动成套设备、技术出口和劳务输出。

（四）扎实开展“项目推进年”活动，千方百计扩大有效投入，提升工业经济综合实力。

****集中开展土地征迁拆违行动。****再好的项目不落地生根、不投产见效，都是一纸空文。把拆迁安置和拆违整治作为事关全局的重要工作，按照“属地管理、以区为主，谁主管、谁负责”的原则，下决心在全市范围内开展大规模集中行动，着力破解项目落地的瓶颈制约。进一步强化县区、开发区主体责任，全面整合配强征迁拆违力量，加强部门协调联动，完善快速高效的工作推进机制，在保障群众合法权益的前提下，全力以赴加快土地征迁和拆违步伐。科学编制土地整治规划，大力推进土地整理，全面开展土地复垦。积极争取土地利用计划和项目单独选址，加大闲置土地清理力度，千方百计满足建设用地需求。进一步明确职责，强化项目落地服务，努力使项目建设周期最短、速度最快、效率最高。

****强力推进重点项目建设。****完善市级领导牵头联系制度，形成领导牵头、部门联动、职责明确、工作到位、督查跟进的项目推进落实机制。及时协调解决续建项目中的突出问题，加快马钢动车组车轮配套、华菱3万辆重卡、立白日用化工二期、方圆重型回转支承生产线等项目建设进度。倒排工期、科学调度，确保泰尔重工联轴器、天成纺织搬迁扩建等项目竣工投产发挥效益。只争朝夕地抓好项目开工，推进一电厂2台60万千瓦机组“上大压小”扩建、科达机电二期、格力电器等项目尽快动工建设。认真落实与央企合作成果，确保签约项目落地生根，建立与央企合作发展的长效机制。落实项目建设支撑要件，推动山鹰80万吨纸板、中钢天源电子科技产业园等重大项目前期工作取得实质性进展。进一步完善项目谋划机制，实行项目动态管理和梯次推进，扩大项目储备规模。高度重视项目与资金的衔接，保障重大项目建设资金需求。力争全年工业性投资增长30%以上。

****促进各类企业加快发展。****支持马钢实施“依托主业、适度多元”发展战略，在提高钢铁主业核心竞争力的同时，积极发展非钢产业，健全应对风险的稳定发展机制，未来五年实现集团年营业收入超1000亿元、非钢产业年产值超150亿元的目标。支持星马、山鹰等重点骨干企业扩张规模，延伸产业链，增强对工业发展的拉动作用。出台扶持壮大行业龙头企业政策措施，精心培育“小巨人”企业，促进专精特新企业加快发展。力争全年新增规模以上工业企业超100户，规模以上工业增加值增长20%以上，销售收入过亿元企业总数超100户。

（五）不失时机地推进改革，构建体制机制新优势，为加快发展增添强大动力和活力。

****深化重点领域和关键环节改革。****巩固市政府机构改革成果，实施县区政府机构改革，切实转变政府职能。深入推进事业单位分类改革，扎实做好事业单位岗位设置和绩效工资改革实施工作。健全经营性国有资产监管体制，把分散沉淀的行政事业单位资产转化成集中流动的国有资本，确保国有资产保值增值。深化文化体制改革，推进广播电视台顺利运营，整合专业文艺团体组建市艺术剧院。深化医药卫生体制改革，扎实开展全国公立医院改革试点，加快推进基层医药卫生体制综合改革试点工作，提前1年实现我省确定的3年改革目标。探索国道省道养护体制改革，启动国有林场改革，深入推进集体林权配套改革。

****加快地方金融体系建设。****健全对金融机构支持地方经济发展的考核激励机制，引导和鼓励银行积极向上争取信贷额度，优化信贷结构，创新和拓展信贷品种，加大对经济社会薄弱环节、战略性新兴产业、中小企业的支持力度，力争全市信贷增量高于去年、增幅高于全省。支持马鞍山农村商业银行增资扩股，开展跨地区设立分支机构和村镇银行，进一步做大做强。加大国内外金融机构引进力度，重点推进光大、民生等银行来我市设立分支机构。新增2家以上小额贷款公司，完成2家以上担保公司增资扩股。按照“上市一批、推进一批、储备一批”的思路，在泰尔重工、鼎泰稀土上市的同时，加快星马汽车重组华菱重卡整体上市步伐，支持奥盛钢缆、惊天液压、华菱西厨等企业做好上市准备，全年实现首发及再融资40亿元以上，着力打造极具活力的“马鞍山板块”。争取20亿元城市建设债券获批发行，并进一步创新融资方式，打造市政建设融资平台，建立更加稳定的融资渠道。充分发挥皖江城市带承接产业转移投资基金功能，再引进发起设立1家创业投资基金。推动股权融资和期货交易，促进保险、证券业发展壮大。

****大力推进全民创业。****扎实开展全国创业型城市试点，进一步完善和落实放宽市场准入、降低创业门槛、结构性减税、企业减负、信贷服务等方面的优惠政策，努力把广大市民的创业愿望变成创业行动。保护民间投资合法权益，注重发挥政府扩大投资对民间投资的拉动作用，大力推动民营企业“二次”创业。加强创业基地建设，完善创业街、创业市场功能，新增创业基地面积15万平方米。坚决清理涉及非公经济和中小企业的不合理收费，进一步降低创业成本。全年新发展个体工商户5000户、私营企业1500户以上。

（六）夯实“三农”基础，扎实推进新农村建设，开创城乡一体化发展新局面。

****下大气力做大做强中心镇经济。****在更大范围实施扩权强镇改革，赋予其部分县级经济社会管理权限，充分调动中心镇加快发展的积极性。坚持特色产业兴镇，大力培育主导产业，重点支持发展一批区位较优、规模较大、层次较高、辐射较强的经济强镇。按照“整合资源、统筹规划、特色发展”的要求，不断提升中心镇规划建设水平，完善公共服务配套设施，努力把中心镇建设成为产业集聚区、人口集中区、服务便利区、和谐社会示范区，着力形成多点支撑的发展格局。放宽户籍限制，积极推进住房、就业和社会保障等制度改革，加快农民市民化步伐。

****大力推进现代农业建设。****加快发展现代农业园区，规划建设现代农产品加工集中区，引导传统农业转型升级，整体提升农业规模化、产业化、高效化水平。重点推进当涂现代农业示范园建设，初步拉开发展框架。加快国家级生猪、奶牛标准化养殖场建设，进一步扩大水产、粮油、蔬菜、苗木花卉基地规模，提升优势农产品市场竞争力。强力推进农业招商引资，培育发展农产品加工企业，推动重点龙头企业不断发展壮大。扶持农民专业合作组织规范发展，支持供销合作社开放办社，不断提高农业组织化水平。加快粮食仓储设施资源整合和建设，继续推进新农村科学储粮示范工程。加强农产品质量安全全程监管，切实抓好重大动植物疫病防控。

****推动城乡一体化发展。****开工建设花山区张庄中心村二、三期项目，实施当涂县、雨山区中心村建设工程。按照依法自愿有偿原则，鼓励农民以转包、转让、出租、互换、股份合作等多种形式流转土地承包经营权，开展土地流转服务平台建设试点，力争土地流转面积增至20万亩。全面完成农村集体土地上房屋确权登记发证工作，进一步规范农村集体土地利用秩序。加强农村中小学标准化建设，在留守儿童较多的乡镇建设寄宿制学校。完成乡镇文化站配套建设，新建40个农家书屋。完善乡镇卫生院基础设施，全面建成标准化村卫生室。完成农村敬老院改扩建任务，力争五保集中供养率达到50%以上。完善农村路网体系，拓展农村客运服务范围。加强农田水利基本建设，提高防灾抗灾能力。加大近郊环卫基础设施投入，加快推进农村生活垃圾和污水集中处理。扩大新农村示范镇、村建设试点范围，放大新农村建设示范带动效应。

（七）大力实施民生工程，健全城乡就业和社会保障体系，努力让人民群众得到更多实惠。

****巩固提升民生工程。****紧紧围绕“基本生活、基本养老、基本医疗、基本住房和困难职工就业再就业、困难家庭子女就学”现阶段民生问题的核心内容，建立框架，逐步覆盖，巩固成果，提升内涵，不断健全和完善民生工程长效机制。进一步加大政府投入，形成稳定、多元的筹资机制，不断扩大民生工程的实施范围，提高民生工程的实施标准和水平。推进校舍安全工程建设，确保全市中小学校舍达到重点设防类抗震设防标准。大规模建设安置房、廉租住房等保障性住房，积极实施危旧房、城中村改造和老旧小区综合整治，增加普通商品房供给。实施标准化光荣院、社会（儿童）福利中心、农村留守儿童之家建设，全力解决群众最关心、最直接、最现实的利益问题。

****努力稳定和扩大就业。****认真落实更加积极的就业政策和困难企业就业帮扶政策，引导劳动密集型企业、中小企业、服务业加快发展，稳定和增加就业岗位。深入推进城乡统筹就业，加快乡镇劳动保障工作平台标准化建设，实施劳动保障信息网络“村村通”工程。积极引导高校毕业生到基层、中小企业就业和自主创业，扶持困难群体通过多种形式灵活就业，鼓励农民工就地就近就业和返乡创业。加强职业院校、技师学院建设，不断提高职业培训水平。整合市人力资源市场，完善统一的公共就业服务制度。全面推进全国劳动保障监察网格化、网络化建设试点工作，切实维护劳动者合法权益，巩固发展和谐劳动关系。全年新增城镇就业2.4万人，新增农村劳动力转移就业1.5万人，完成职业技能培训1万人次。

****进一步完善城乡社会保障制度。****支持养老保险省级统筹，实现城镇企业职工基本养老保险关系可转移接续，进一步扩大参保面。扎实开展全国新农保试点，全力推进当涂县新农保参保扩面达标。开展居民生育保险全国试点，完善现行生育保险政策，出台灵活就业人员和个体工商户生育保障办法，探索建立城乡一体的生育保险制度。健全城乡居民医疗保险政策，完善失业保险、工伤保险市级统筹制度。加强社会保险费征缴稽核，确保基金安全和社会保险金按时足额发放。全面实施城乡居民最低生活保障制度，强化城乡最低生活保障动态管理。健全城乡一体的医疗救助制度，提高医疗救助上限标准。积极开展居家养老和社区养老服务，提升社会化养老服务水平。

（八）坚持以人为本，加快发展各项社会事业，着力提高公共服务和社会管理水平。

****优先发展教育。****推进义务教育均衡发展，规范中小学办学行为，全面提高义务教育质量，促进教育公平。进一步调整普通高中布局，完成南部高中主体工程建设，推动普通高中优质化发展。全面实施职业教育现代化工程，推进中职集团和中职园区发展，加强实训基地和骨干专业建设，力争全市高中阶段教育毛入学率达到88%以上。推进安工大东校区二期、河海大学文天学院二期工程建设，开展省示范高职高专创建工作，促进地方高等教育提升发展。

****推动文化繁荣发展。****加强社会主义核心价值体系建设，积极发展广播影视、新闻出版、文学艺术和哲学社会科学，深入实施全民科学素质行动计划纲要，大力营造和弘扬健康向上的文化氛围。加快文化产业发展，培育一批有竞争力的文化龙头企业。推进文化保护工程，完成第三次全国文物普查，申报太白楼为全国重点文物保护单位。实施文化精品工程，高水平承办第九届安徽省艺术节，办好’2010马鞍山中国李白诗歌节，积极申办第三届中国动漫节。完善公共文化服务体系，确保大剧院按期建成运营，实施市文化馆改扩建工程，开工建设市体育中心和集科技馆、青少年宫、妇女儿童活动中心为一体的综合馆。启动档案电子文件中心暨文档一体化项目建设，进一步做好地方志工作。深入开展全民健身活动，积极备战省第十二届运动会。

****提升医疗卫生服务能力。****建成人民医院门诊大楼、传染病医院门诊楼并投入使用，加快中医院南迁工程步伐，启动人民医院外科综合大楼、妇幼保健院综合住院楼建设工程，逐步改善人民群众就医环境。合理配置医疗资源，加强医疗质量管理，完善医疗监管体系。坚持鼓励支持与监督管理并重，推动民营医疗机构健康发展。健全城乡基层医疗卫生服务体系，完成社区卫生服务中心建设。加强卫生人才队伍建设，改善医疗服务质量，提高医疗技术水平。扩大公共卫生服务项目和免疫规划实施范围，抓好重大传染病、地方病及人畜共患病防治。认真做好第六次全国人口普查工作，全面推进人口计生综合改革，稳定低生育水平。

****维护社会和谐稳定。****深入推进平安马鞍山建设，强化社会治安综合治理，着力加强以视频监控和大情报平台建设为主的公安信息化建设，规范执法行为，和谐警民关系，夯实公安基层基础工作，严厉打击各类犯罪行为。继续开展为企业和重点项目“保平安”活动，稳步推进以“零发案小区”为目标的平安小区建设，扎实开展禁吸戒毒工作。认真做好社区矫正、刑释解教人员安置帮教和法律援助工作，加强基层司法所建设，发挥人民调解基础性作用。推进大信访接待中心项目建设，加强和改进信访工作，最大限度化解不和谐因素。积极创建精品、四优社区，巩固提升村务公开和民主管理水平。充分发挥工会、共青团、妇联等人民团体的桥梁纽带作用，扶持和规范各类社会组织发展，支持其参与社会管理和公共服务。大力整顿和规范市场秩序，加强食品药品安全日常监管和专项整治。进一步强化安全生产和安全监管主体责任，严格落实规章制度，促进安全生产形势持续稳定。健全完善应急管理工作体系，进一步提高应对突发事件和保障公共安全的能力。推动民族团结进步事业创新发展，发挥宗教在促进社会和谐方面的积极作用。重视发展老龄事业，切实保障妇女、儿童和未成年人合法权益，逐步健全残疾人服务保障体系。加强公共气象、防震减灾工作。积极支持驻马部队建设，完善民兵训练基地功能，做好国防动员、民兵预备役和人防工作，提升双拥优抚安置水平，巩固发展军政军民团结。

****三、进一步加强政府自身建设****

复杂的经济形势，繁重的发展任务，对政府工作提出了更高的要求。我们唯有不断加强政府自身建设，切实提高施政水平和行政效率，把各项发展措施谋在先处、落到实处，才能肩负起时代和人民赋予的重任。

****坚持创新推动，增强科学发展本领。****把强化学习作为改革创新的先导，扎实推进学习型政府建设，努力学习履行职责必需的知识，掌握适应新形势、新任务、新要求的本领，着力提高战略思维、创新思维和辩证思维能力。始终保持强烈的发展意识，多思发展之法，多谋发展之策，多出发展之力，多求发展之效。更加重视发展方式的优化和创新，绝不停留在原有增长方式的延续和复苏上，全力追求发展的更高质量和更好效益。大力营造创新环境，敢走新路，善破难题，鼓励探索，宽容失败。着力推进工作体制机制创新，摒弃对抗性思维，增强适应性才干，做到既有想法、更有办法，既会出题、更会做题，在有想法、有办法中推动发展，在出题、做题中创造业绩。高度重视“十二五”规划编制工作，创新规划理念和方法，凝聚各方智慧和力量，切实增强规划的预见性、科学性和指导性，努力以更快速度、更高水平把坚持科学发展、走在全省前列的宏伟大业不断推向前进。

****提振精神状态，保持干事创业激情。****良好的精神状态是克服困难、夺取胜利的首要前提，也是我们应对危机、力保增长的深刻体会。完成今年各项任务、继续走在全省前列，对我们的精神状态和工作作风提出了新的更高要求。要切实增强等不起的紧迫感、慢不得的危机感、坐不住的使命感，以敢想敢闯的宏伟气魄、敢做敢干的拼搏精神、敢为敢当的责任意识，做到复杂局面善于驾驭、重大问题勇于担当、关键时刻敢于负责。无论遇到什么困难和挑战，都要坚定信心，凝心聚力，不动摇，不折腾，不懈怠，按认准的科学发展之路走下去。把政府追求的目标和老百姓的期盼统一起来，坚决克服形式主义、官僚主义，全心全意为发展服务、为基层服务、为群众服务。政府系统各级领导干部尤其要坚持重心下移，深入问题困难多、群众意见多、工作瓶颈多的地方，办好顺民意、解民忧、惠民生、促发展的实事好事。

****强化执行落实，着力提升行政效能。****任何好的规划和设想，如果缺乏实干，只能是空中楼阁。坚持事业为上、责任为重、落实为要，不说空话，不避难题，多干实事。健全保障落实的工作机制，明确工作的目标举措、时限要求、责任主体，把任务一项一项抓实，把事情一件一件办好。健全目标管理责任体系，创新目标绩效考核评价机制，进一步加大政务督查力度，做到有令必行、有禁必止、言出法随，确保政令畅通。结合市县政府机构改革，深入推进机关效能建设，加大治懒治庸力度，落实首问负责制、限时办结制，提倡立即办、马上办、办得快、办得好。继续深化行政审批制度改革，进一步减少审批项目，优化审批流程，扩大并联审批范围，积极推行网上办事、网上审批和电子监察。加强电子政务建设，进一步降低行政成本，提高工作效率，努力营造办事最快、成本最低、服务最优的政务环境。

****推进依法行政，努力规范施政行为。****坚持市委的统一领导，依法履行政府经济调节、市场监管、社会管理、公共服务职责。自觉接受市人大法律监督、工作监督和市政协民主监督，高质量地办理人大议案、建议和政协提案。认真听取各民主党派、工商联、无党派人士及各人民团体的意见，主动接受社会公众和新闻舆论监督。全力推进法治政府建设，认真落实依法行政各项制度，创新行政管理方式。规范行政执法行为，充分发挥行政复议的救济与监督功能。推进依法决策制度建设，健全公众参与、专家论证和政府决策相结合的决策机制，切实保证决策的科学化、民主化和透明化。深入推进政务公开，实现政务公开全覆盖。进一步办好市长公开电话、政风行风热线和政府网站，广开渠道听取意见和建议，不断改进政府工作。

****加强廉政建设，树立清正廉洁形象。****深入推进政治生态建设，健全完善教育、制度、监督并重的惩治和预防腐败体系，认真落实党风廉政建设责任制和领导干部廉洁自律预警机制。加强对领导干部、人财物管理使用、关键岗位的监督和制约，防止权力失控、决策失误、行为失范。完善全市集中统一的招标采购监管体制，进一步规范招标采购行为，深入开展工程建设领域突出问题专项治理，努力从源头上预防和治理腐败。进一步加强对新增中央投资项目实施的监督检查，严格执行离任审计、离任与接任经济事项交接、重大事项报告等制度。按照节俭、高效、廉洁的原则，大力削减行政成本，把有限的资金和资源更多地用在发展经济、改善民生上。全体政府工作人员特别是领导干部一定要勤政廉政、自警自励，堂堂正正做人，清清白白做事，努力树立为民、务实、清廉的政府形象。

各位代表，新的一年任务艰巨而繁重，责任重大而光荣。让我们高举中国特色社会主义伟大旗帜，在省委、省政府和市委的正确领导下，紧紧依靠全市人民，同心同德，顽强拼搏，推动全市经济社会高质量、大体量、快速度发展，为坚持科学发展、走在全省前列、早日把马鞍山建设成为具有时代特色的现代化大城市而努力奋斗！
